# Single Cell Transcriptome Sequencing of Zebrafish Testis Revealed Novel Spermatogenesis Marker Genes and Stronger Leydig-Germ Cell Paracrine Interactions

**DOI:** 10.3389/fgene.2022.851719

**Published:** 2022-03-11

**Authors:** Peipei Qian, Jiahui Kang, Dong Liu, Gangcai Xie

**Affiliations:** ^1^ Institute of Reproductive Medicine, Medical School, Nantong University, Nantong, China; ^2^ School of Life Sciences, Key Laboratory of Neuroregeneration of Jiangsu and Ministry of Education, Co-innovation Center of Neuroregeneration, Nantong University, Nantong, China

**Keywords:** single cell, testis, spermatogenesis, zebrafish, transcriptome, Leydig cell, paracrine

## Abstract

Spermatogenesis in testis is an important process for sexual reproduction, and worldwide about 10–15 percent of couples suffer from infertility. It is of importance to study spermatogenesis at single cell level in both of human and model organisms. Currently, single-cell RNA sequencing technologies (scRNA-seq) had been extensively applied to the study of cellular components and its gene regulations in the testes of different species, including human, monkey, mouse, and fly, but not in zebrafish. Zebrafish was a widely used model organism in biology and had been extensively used for the study of spermatogenesis in the previous studies. Therefore, it is also important to profile the transcriptome of zebrafish testis at single cell level. In this study, the transcriptomes of 14, 315 single cells from adult male zebrafish testes were profiled by scRNA-seq, and 10 cell populations were revealed, including Leydig cell, Sertoli cell, spermatogonia cell (SPG), spermatocyte, and spermatids. Notably, thousands of cell-type specific novel marker genes were identified, including *sumo3b* for SPG, *krt18a.1* for Sertoli cells, *larp1b* and *edrf1* for spermatids, which were also validated by RNA *in situ* hybridization experiments. Interestingly, through Ligand-Receptor (LR) analyses, zebrafish Leydig cells demonstrated stronger paracrine influence on germ cells than Sertoli cells. Overall, this study could be an important resource for the study of spermatogenesis in zebrafish and might also facilitate the study of the genes associated with human infertility through using zebrafish as a model organism.

## Introduction

In animals, testis is the primary male reproductive organ that generates sperms for sexual reproduction. It had been estimated that 10–15% of couples worldwide suffer from infertility, and male factors attribute to about 20–30% of all factors leading to infertility (Babakhanzadeh et al., 2020). Spermatogenesis in testis is an important process to generate sperms through undergoing meiosis I and II, which involves a sequential of cell types, such as spermatogonia stem cells (SSC), spermatocytes derived from SSCs through mitotic cell division, haploid spermatids through meiotic cell division, and the supporting somatic cells (eg., Leydig cell and Sertoli cell) (Nishimura and L’Hernault, 2017).

The advent of single-cell RNA sequencing (scRNA-seq) has greatly enlarged our understanding of the transcriptome landscape for various organs in different species. During past years, scRNA-seq had been used to characterize cell heterogeneity and identify novel cell types in various studies, including studies for development, cancer, and kidney diseases (Potter, 2018). Notably, scRNA-seq had been widely used to study spermatogenesis in mammals, such as the studies of murine spermatogenesis (Chen et al., 2018; Green et al., 2018; Lukassen et al., 2018; Grive et al., 2019; Jung et al., 2019), human testis single-cell level studies for different age stages (Guo et al., 2018; Sohni et al., 2019; Guo et al., 2020), and single-cell level evolutionary studies in macaques (Lau et al., 2020; Shami et al., 2020).

In contrast to mammals, due to lacking sex chromosomes, the sex determination and timing of testis differentiation is different in zebrafish (Orban et al., 2009). Besides, zebrafish testis is composed by cystic structure for spermatogenesis (Schulz et al., 2010), while in mammals non-cystic structure seminiferous tubule is the basic units for spermatogenesis. Although there are several studies (Chen et al., 2018; Green et al., 2018; Guo et al., 2018; Lukassen et al., 2018; Grive et al., 2019; Jung et al., 2019; Sohni et al., 2019; Guo et al., 2020; Lau et al., 2020; Shami et al., 2020) for mammalian testis at single-cell level, it is still lacking for the study of zebrafish testis by scRNA-seq.

In this study, we applied 10X genomics scRNA-seq technology to the study of pooled adult zebrafish testes, and profiled the transcriptome of 14, 315 single testicular cells. In total, we identified 10 distinguishable cell types in zebrafish testis, including four types of Spermatids, one type of Spermatocyte, two types of Spermatogonia cells (SPG), two known somatic supporting cells (Leydig and Sertoli cells). Besides identification of basic cell types in zebrafish testis, novel marker genes were also revealed and experimentally validated by this study. Furthermore, through Ligand-Receptor (LR) analyses, more LR interactions were found between somatic cell and germ cell in Leydig than in Sertoli, which indicates stronger paracrine influence from Leydig cell than from Sertoli cell in zebrafish testis. Overall, our study might be a valuable resource for spermatogenesis studies in zebrafish testis.

## Materials and Methods

### Sample Preparation and Single Cell RNA Sequencing

5 month-old adult AB line zebrafish (*Danio rerio*) were prepared and treated with tricaine methanesulfonate (MS-222, 0.25%) on ice for 15 min before experiment. The study was conducted in line with the Chinese law for the Protection of Animals, and the animals were treated properly. The testes samples collected from those zebrafish were first washed by PBS three times, and then were digested in 10 ml 0.25% trypsin at 37°C for 15 min, during which process the tissues were pipetted up and down every 3 min. The cell suspension was first filtered through 70 μm nylon mesh after stopping the digestion process by DMEM (with 10% FBS), and then were centrifuged at the speed of 1,000 rpm for 10 min. After centrifugation, the cells were suspended in 1 ml DMEM (with 10% FBS) and were further filtered through 40 μm nylon mesh. Before loading onto the 10x chromium chip, the cells were washed by BSA DPBS (0.04%, three times) and were resuspended to a concentration of 800∼1,000 cells/μl (viability >85%). The single cell library preparation was carried out according to the manufacturer’s protocol (Chromium Single Cell 3′ Reagent Kits V3 Chemistry). The cDNA library was further size filtered and sequenced on the Illumina NovaSeq 6,000 System following 150 bp paired-end sequencing protocol.

### ScRNA-Seq Analysis

Cell ranger software (v5.0.0) from 10X genomics was used to preprocess the raw sequencing data, and primary UMI filtering was carried out by Cell ranger following criteria “No N contained in the UMI,” “all base quality should be no less than 10,” and “not a homopolymer sequence.” For cell ranger processing, zebrafish genome and gene annotation from Ensembl were used as reference datasets (Ensembl Gene 102). Then, R package Seurat (V4.0.0) was used for further data filtering, data normalization, cell clustering, and cluster-level marker gene discovery. The UMI matrix data was filtered at two dimensions: gene filtering and cell filtering. For gene filtering, only the genes that were detected at least in 5 cells were retained. For cell filtering, the retained cells should contain the number of expressed genes within the range between 200 and 5,000, and the proportion of UMIs from mitochondria should be less than 5%. The raw UMI matrix was normalized by sctransform, which fitted the data through regularized negative binomial regression. Three types of features were used for data regression: total number of expressed genes, total number of UMIs, and the percentage of UMIs from mitochondria. In order to cluster cells, the principal components (PC) were computed by principal components analysis (PCA), and the first fifteen PCs were used for cluster finding. K nearest neighbors (KNN) method was applied to cell clustering, where the K was set to be 20. Finally, the marker genes for each cell cluster were statistically computed by Wilcoxon Rank Sum test, and only the genes detected in at least 25% of the cells (in either of the two tested cell clusters) were included for testing. The marker genes were defined as the genes with *p* value less than 0.01 and the log transformed (base 2) fold change larger than 0.25.

To annotate the cell clusters identified in this study, the testicular cell-type marker genes were collected from previous studies in zebrafish (Leal et al., 2009; Chen et al., 2013; Assis et al., 2016; Safian et al., 2016; Lin et al., 2017; de Castro Assis et al., 2018; Takemoto et al., 2020) or other species (Hermann et al., 2018). In the further analyses, only the collected marker genes showing cell-type specific expression patterns in our single cell studies were used for annotation. The final list of known marker genes and their sources can be found in Supplementary Table S1.

### Ligand-Receptor Based Cell-Cell Communication Analyses

In order to study the cell-cell communication among cell populations, the Ligand-Receptor (LR) pairs were analyzed, which followed the procedure with minor modifications described in one recently published paper (Shi et al., 2021) about LR studies in mouse. In detail, CellTalkDB (Shao et al., 2020) was selected as the database for LR analyses, which contained 3,398 manually curated human LR pairs. The orthologous genes between human and zebrafish were downloaded from Ensembl biomart (Ensembl Genes 102), and then 13,004 one-one orthologous genes were retained for LR analyses. SingleCellSignalR (Cabello-Aguilar et al., 2020) was applied to the inference of intercellular LR interaction networks, where the default LR database was replaced by CellTalkDB and only the genes found in the zebrafish-human one-one orthologous gene list were included for the analysis.

### H&E Staining

Adult zebrafish (5 months after fertilization) were anesthetized in 0.25% ms-222 (ethyl 3-aminobenzoic; Sigma, E10505) in filtered system water. Zebrafish testes were fixed with 4% paraformaldehyde (Sigma, P6148) overnight at 4°C. Then, the fixed samples were embedded in OCT (Sakura, 4583) after dehydration of sugar, sectioned at 10 μm thickness, and stained with hematoxylin and eosin (BBI, E607318).

### RNA *in situ* Hybridization

Probe fragments were first amplified from zebrafish testes cDNA libraries (primers information in Supplementary Table S2) and then inserted into pGEM-T-easy vector (Promega, A1360). Digoxigenin-labeled antisense RNA probes were made by using DIG-RNA labeling Kit (Roche, 11175025910). Zebrafish testes were cut into 12 μm slices by cryotome under temperature between −25°C and −23°C. Slides were first fixed with 4% PFA, then digested with 0.1% Proteinase K, followed by washing in PBS. Lastly, the digested slides were first incubated with the digoxygenin-labelled RNA probes at 68°C overnight and then incubated with alkaline phosphatase-conjugated anti-DIG antibody, followed by treatment with AP-substrate NBT/BCIP solution (Roche, 11681451001) for visualization. Brightfield images of sections were obtained with Nikon Eclipse Ni-U microscope.

### Data Visualization and GO Enrichment Analyses

To visualize the cell clusters and the gene expression patterns in two-dimensional graphs, Uniform Manifold Approximation and Projection (UMAP) was applied to dimensional reduction, where the first 15 PCs from PCA were used as the input for UMAP. ClusterProfiler (Yu et al., 2012) (version 3.18.0) was used for GO enrichment analyses, and the *p* values were corrected by Benjamini-Hochberg (BH) method. Only the GO terms with BH corrected *p* value less than 0.05 were retained as significant ones.

### Data and Software Availability

The raw sequence data reported in this paper had been deposited in the Genome Sequence Archive (Wang et al., 2017) in National Genomics Data Center (Members, 2021), China National Center for Bioinformation/Beijing Institute of Genomics, Chinese Academy of Sciences, under accession number CRA003925 that are publicly accessible at https://bigd.big.ac.cn/gsa. All the codes used for this study can be found in https://github.com/gangcai/zebTestis.

## Results

### Single Cell Transcriptome Profiling of Zebrafish Testis

**FIGURE 1:**
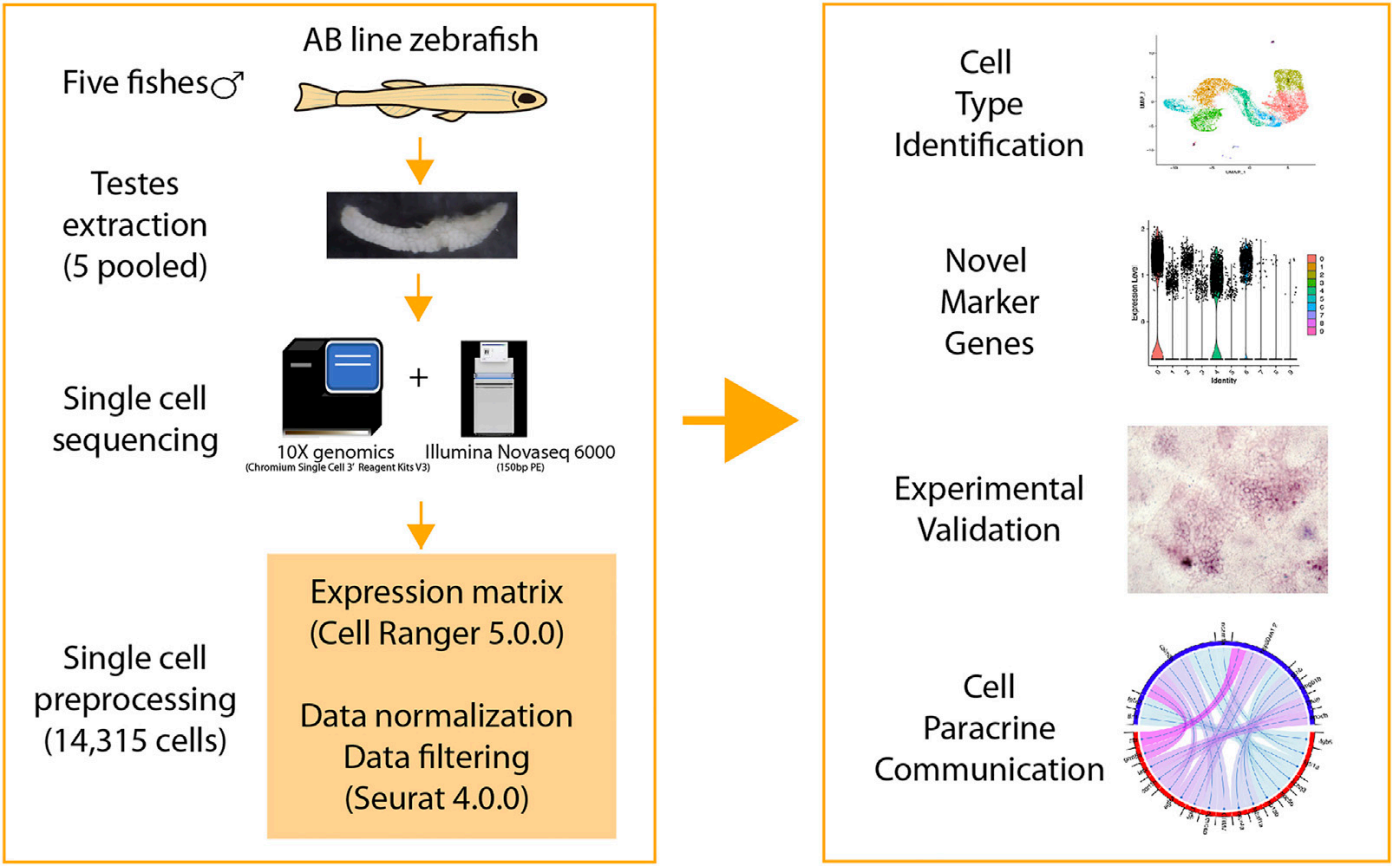
Overview of this study.

**FIGURE 2:**
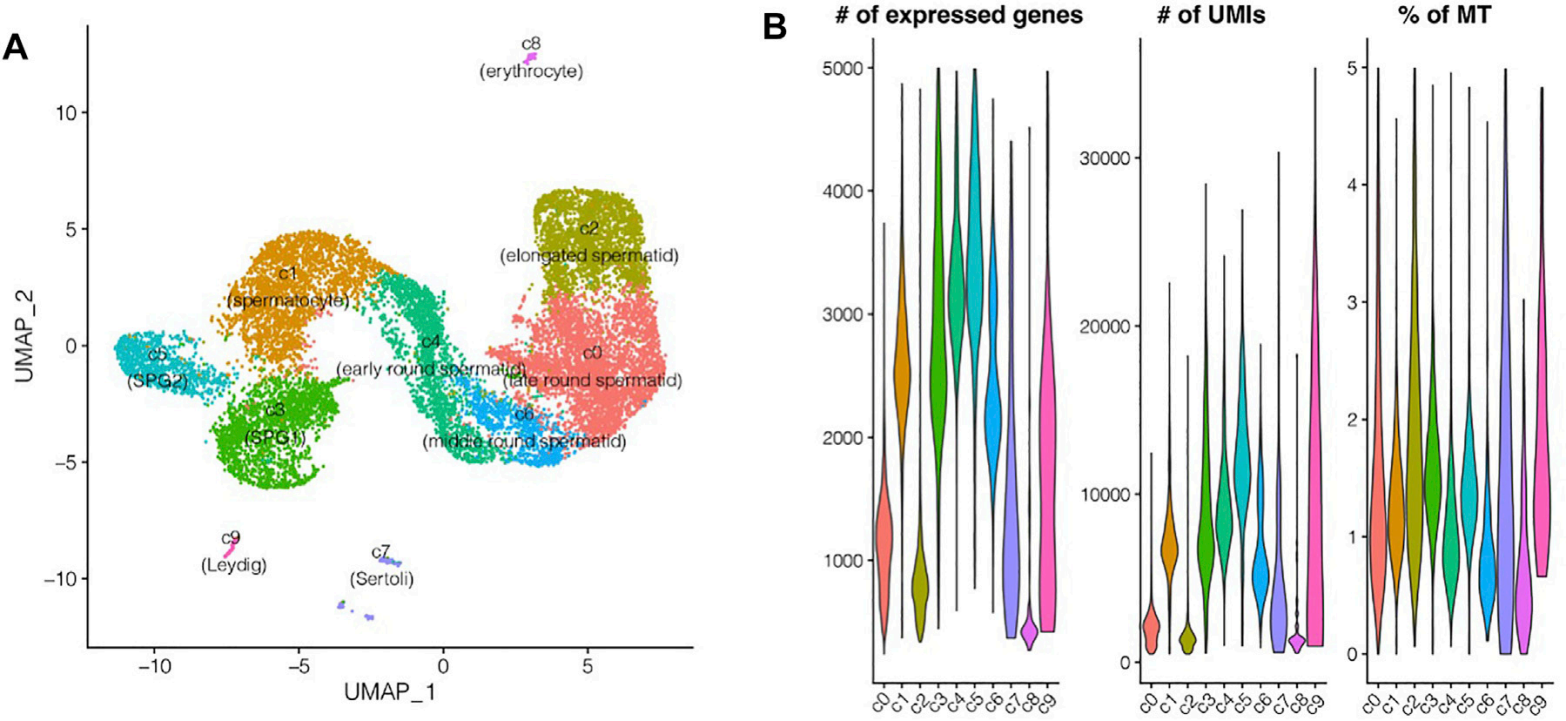
Single cell RNA sequencing of zebrafish testes. **(A)** UMAP representation of 10 cell populations. **(B)** Violin plots for the distribution of the number of expressed genes, UMIs, and the percentage of mitochondrial UMIs.

Testes from five adult male zebrafish were pooled for 10X genomics single RNA sequencing, initially there were 16,032 single cells detected, and after quality filtering 14,315 single cells were remained and used for further analyses (Figure 1; Supplementary Figure S1A). 10 different cell types were identified, including spermatogonia subpopulation 1 and 2 (c3, c5), spermatocyte (c1), early round spermatid (c4), middle round spermatid (c6), late round spermatid (c0), elongated spermatid (c2), Leydig (c9), Sertoli (c7), and a small proportion of red blood cells (c8) (Figure 2A). In detail, the cell type with highest proportion was late round spermatid, and the second highest one was spermatocyte, which were 3,889 and 2,396 single cells respectively (Supplementary Figure S1B). Notably, the proportion of two somatic cells (Leydig and Sertoli) is relatively small compared to the germ cells, in total, we identified 58 Leydig cells and 133 Sertoli cells (Supplementary Figure S1B). The top three cell types with largest number of detected genes are SPG2, early round spermatid and SPG1, and the average number of genes is also positively corrected with the average number of UMIs (Figure 2B; Supplementary Figures S1C,D). Lower gene expression was detected in late-stage cell types of spermatogenesis (elongated spermatids, and late round spermatids) (Supplementary Figure S1D). All the cell types identified contain lower proportions of expressed mitochondrial genes (0.5–2% on average, Figure 2B), which indicates rare contamination of apoptotic, stressed, or lower quality cells.

The cell types were annotated based on known marker genes examined in zebrafish testis or the testis of other species (Supplementary Table S1; Supplementary Figure S2). For example, *star* was reported as the marker gene of Leydig cell (Lin et al., 2017), and our scRNA-seq data clearly showed that it was specifically expressed in cell cluster c9. Furthermore, based on previous studies, *sycp2* had been recognized as a marker gene for spermatocyte cells (Takemoto et al., 2020), while *dazl* was mainly expressed in SPG and weakly detected at later spermatogenesis stages (Chen et al., 2013). In this study, *sycp2* was found to be significantly enriched in c1, and *dazl* was discovered to be highly enriched in c3 and c5. Supplementary Table S3

### Novel Zebrafish Spermatogenesis Marker Genes

**FIGURE 3:**
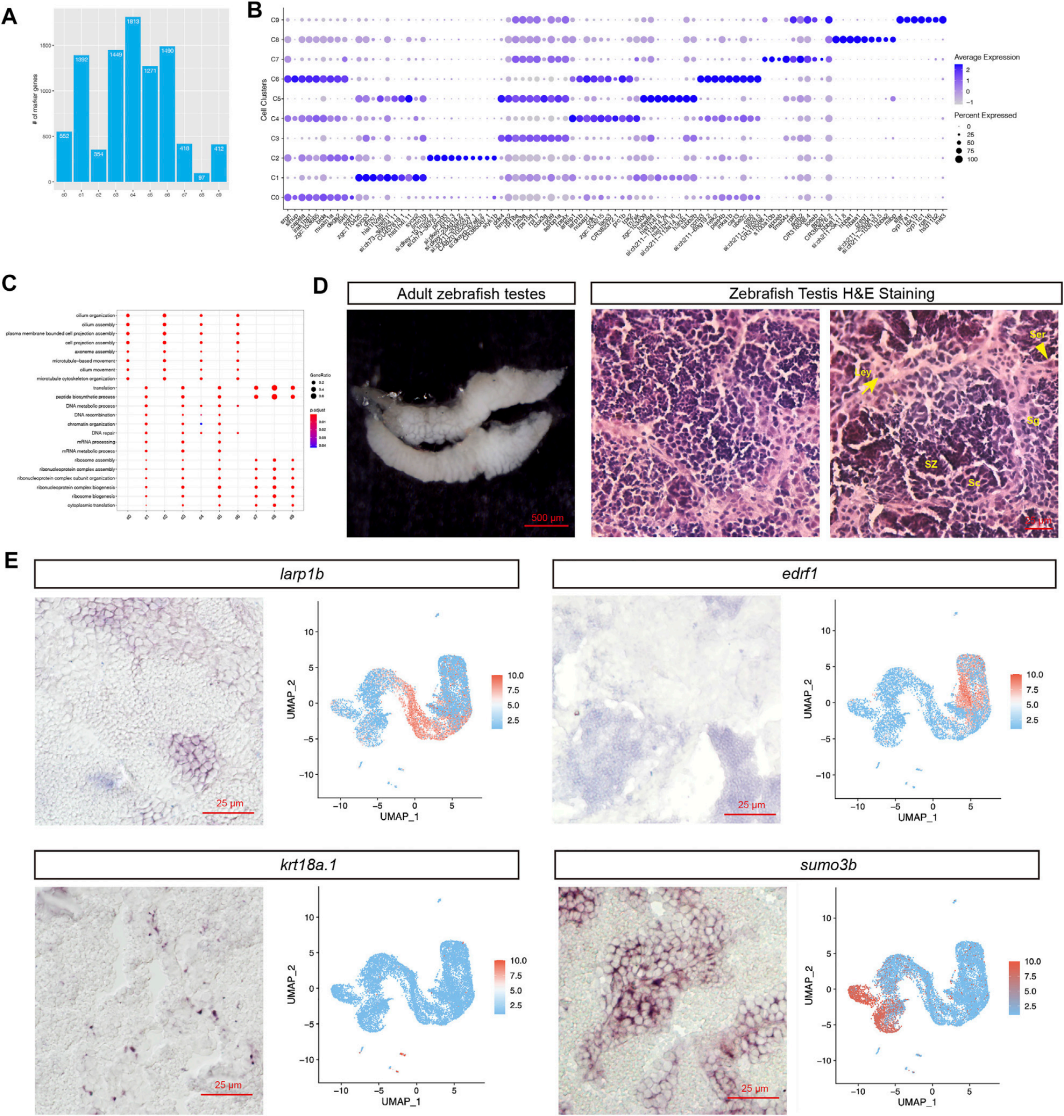
Studies of novel marker genes for each cell population. **(A)** Distribution of the number of marker genes in each cell population. **(B)** Dotplot visualization of top 10 marker genes for each cell cluster. **(C)** GO term enrichment analysis of the marker genes. **(D)** H&E staining of different cell types (Ser, Sertoli cell; Sg, Spermatogonia; Sc, Spermatocyte; SZ: spermatozoa; Ley: Leydig cell). **(E)** RNA *in situ* hybridization of novel marker genes. (For each marker gene, both of the ISH experimental result (Left) and scRNA-seq UMAP representation of gene expression were presented (Right))

Next, the cluster level marker genes were examined, in total, thousands of marker genes were identified for each cell type, including 1,449 marker genes for SPG1, 1,271 marker genes for SPG2, 418 genes for Sertoli cells, and 412 genes for Leydig cells (Figure 3A; Supplementary Table S3). The top 10 marker genes based on the rankings of gene expression foldchange can be found in Figure 3B, including *star* for Leydig (c9), *sycp2* for Spermatocyte (c1), *hbba1*/*hbba2* for red blood cell (c8).Based on the marker genes, GO enrichment analysis was performed: “cilium assembly,” “cilium movement,” “cilium organization” GO terms were significantly enriched in spermatids (c4, c6, c0, c2), and “DNA recombination,” “chromatin organization,” “mRNA metabolic process” GO terms were significantly enriched in early-stage cell types of spermatogenesis (c1, c3, c5) (Figure 3C). Notably, “ribosome assembly” and other ribosome related terms were enriched in non-spermatids cell types, which might indicate lack of translation events in spermatids.

The cluster-level marker genes provide a way to find novel spermatogenesis marker genes. We found *edrf1* (erythroid differentiation regulatory factor 1) was specifically expressed at elongated spermatids, and *larp1b* (La ribonucleoprotein 1B) RNA was enriched in round spermatids (Figure 3B). We further carried out RNA *in situ* hybridization (ISH) to validate the novel testicular cell type specific marker genes. Firstly, for better recognition of each cell types in zebrafish testis, we annotated the cell types based on the cell shapes and relative locations in the H&E staining image (Figure 3D). As we expected, the ISH staining for *larp1b* and *edrf1* RNA showed positive signal in round spermatids and elongated spermatids respectively, and the ISH staining for *krt18a.1* and *sumo3b* showed positive signal in Sertoli and Spermatogonia (SPG) cells respectively (Figure 3E). Furthermore, we also provided all the novel marker genes for each zebrafish testicular cell type in Supplementary Table S3.

### Subpopulation Study of Spermatogonia Cells Identified Marker Genes for SPG1 and SPG2

**FIGURE 4:**
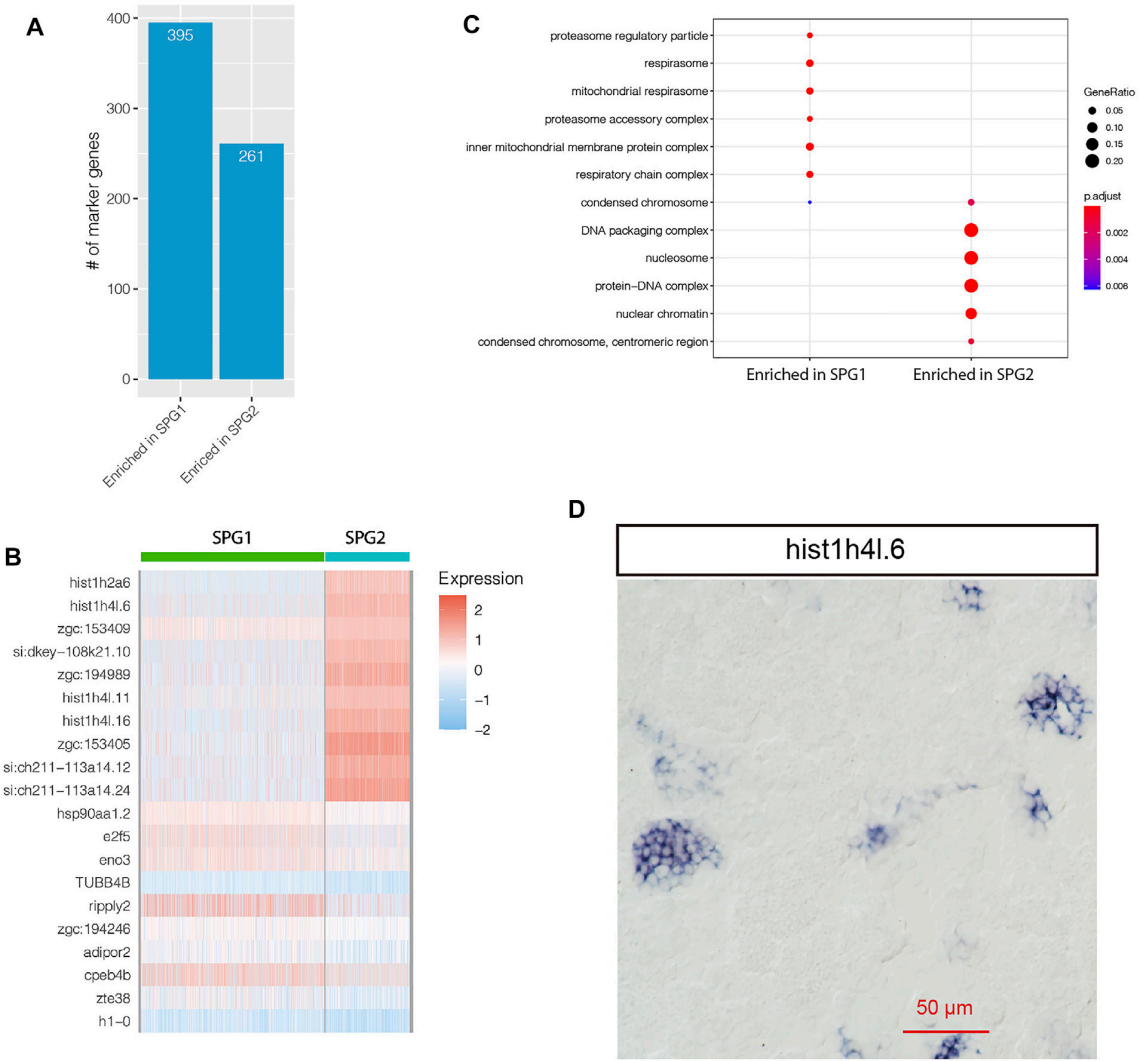
Comparative study of SPG1 and SPG2. **(A)** Distribution of the number of genes enriched in SPG1 or SPG2. **(B)** Heatmap visualization of top 10 marker genes. **(C)** GO term enrichment analysis of the enriched genes. **(D)** RNA *in situ* hybridization result for SPG2 specifically expressed gene hist1h4l.6.

For zebrafish spermatogonia cells, two subpopulations were found in this study: SPG1 and SPG2 (Figure 2A). In total, 656 genes were significantly differentially expressed between SPG1 and SPG2 (Figure 4A, Supplementary Table S4). We found histone related genes involved in heterochromatin assembly were enriched in SPG2, such as *hist1h2a6*, *hist1h4l.6*, *hist1h4l.11* (Figure 4B). Further GO enrichment analysis based on SPG subpopulation specifically expressed genes confirmed that the GO terms such as “condensed chromosome,” “DNA packaging complex” were significantly enriched in SPG2 (Figure 4C). In human, there were three types of spermatogonia: Type A dark, Type A pale, and Type B, where type A pale went through division every seminiferous epithelial cycle (Kolasa et al., 2012). In zebrafish, there were also three types of spermatogonia: type A undifferentiated (A_und_), type A differentiated (A_diff_), and type B spermatogonia (Schulz et al., 2010). The enrichment of GO term “condensed chromosome” suggests more histone proteins involved for chromosome DNA packaging, as shown in Figure 4B histone protein genes such as *hist1h2a6*, *hist1h4l.16* were significantly enriched in SPG2. Further ISH experiment for *hist1h4l.6* RNA confirmed its highly specific expression pattern in spermatogonia cells (Figure 4D). The enriched GO terms and high expression level of histone protein genes in SPG2 suggests that SPG2 was actively undergoing mitosis and differentiation, which was in line with the definition of type A differentiated spermatogonia in zebrafish (A_diff_) and type B spermatogonia. In contrast to SPG2, SPG1 was not enriched with histone protein genes and DNA packaging complex (Figure 4C), which indicates that SPG1 was not actively involved in mitosis, and it might be the type A undifferentiated spermatogonia cells (A_und_). New marker genes for A_und_ were found based on our scRNA-seq datasets, such as *eno3*, *e2f5*, and *ripply2* (Figure 4B). Interestingly, *ENO3* gene might be involved in muscle regeneration, and had been recognized as one of the marker genes during muscle regenerative phase of maturation (Forcina et al., 2020). Furthermore, *E2F5* gene belongs to *E2F* family of transcription factors, and it might play a role as a tumor suppressor gene and control the cell cycle process (Chen et al., 2009).

### Leydig Cells Manifest Stronger Paracrine Influence on Germ Cells

**FIGURE 5:**
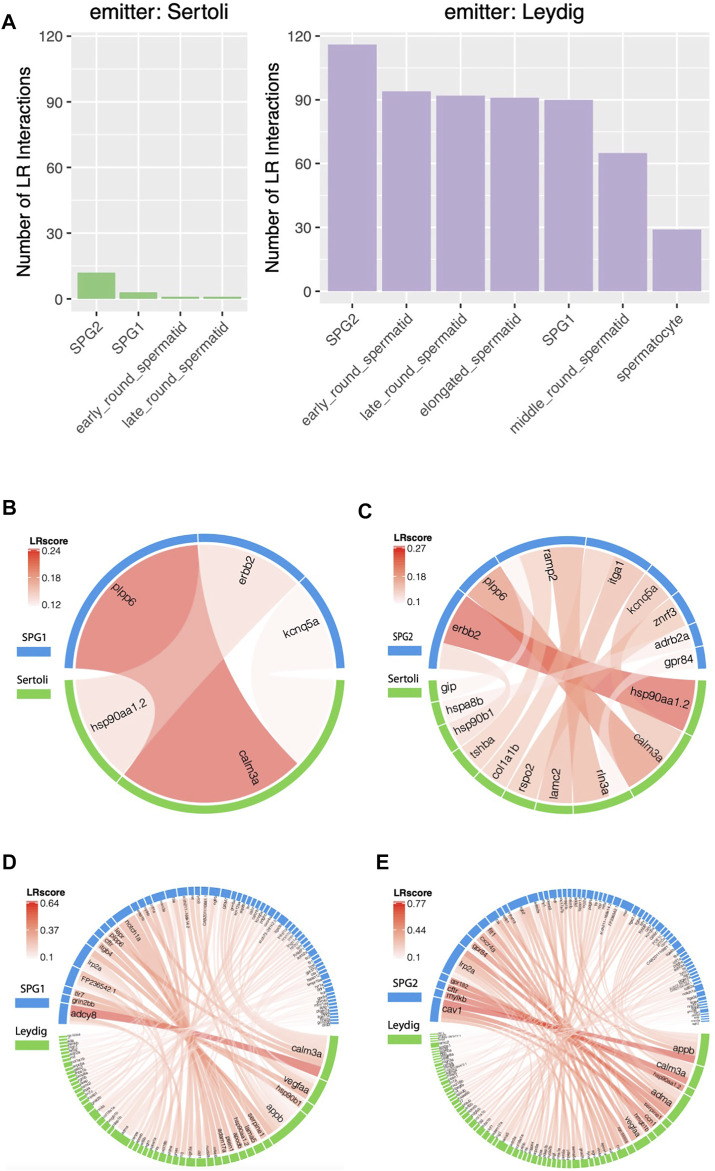
Comparative studies of paracrine influence of Leydig and Sertoli cells. **(A)** Summary of the Ligand-Receptor (LR) interactions between somatic and germ cells. **(B)** LR interaction chord diagram for the influence of Sertoli cells on SPG1. **(C)** LR interaction chord diagram for the influence of Sertoli cells on SPG2. **(D)** LR interaction chord diagram for the influence of Leydig cells on SPG1. **(E)** LR interaction chord diagram for the influence of Leydig cells on SPG2.

Lastly, we studied the influence of somatic cells on zebrafish germ cells and the spermatids. As shown in Figure 5A, more LR interactions were observed between Leydig-Germ cells than Sertoli-Germ cells, which demonstrated a stronger influence of Leydig cell on germ cells than Sertoli cells. However, based on previous studies, Sertoli cell played important roles in germ cell differentiation, survival, development, and physiological functioning (Schulz et al., 2010), which made us expect more LR interactions emitted from Sertoli than Leydig. In our study (Figure 5A), both of the two somatic cells (Sertoli and Leydig, as LR interaction signal emitter) had most abundant paracrine interactions with SPG2 (as LR interaction signal receiver), while the number of LR interactions emitted from Leydig (116) is much more than the number of LR interactions emitted from Sertoli (12). Among two spermatogonia cells, both of Leydig and Sertoli cells exhibit higher influence on SPG2 than SPG1. The detail LR interactions between somatic-SPG cells were shown by chord diagrams (Figures 5B–E). *Calm3a*-*plpp6* was predicted with highest LR interaction score (LRscore) between Sertoli (emitter) and SPG1 (receiver) (Figure 5B), and *hsp90aa1.2*-*erbb2* showed the strongest paracrine interaction between Sertoli and SPG2 (Figure 5C). The top LR interactions between Leydig and SPG1 include *calm3a*-*adcy8*, *vegfaa*-*grin2bb*, *hsp90b1*-*tlr7*, *appb*-*FP236542.1* (Figure 5D), and the top LR interactions between Leydig and SPG2 include *appb*-*cav1*, *calm3a*-*mylkb*, *hsp90aa1.2*-*cftr*, *adma*-*gpr182* (Figure 5E). We also analyzed other paracrine interactions between each testicular cell, and the full list of LR interactions and their LR interaction scores can be found in Supplementary Table S5. Our result based on the comparative analyses of the paracrine interactions between somatic-germ cells suggested the importance for the future study of the paracrine influence of Leydig on Germ cells.

## Discussion

In this study, for the first time, the whole transcriptome of the zebrafish testis was sequenced at single cell level. In total, the transcriptome information of 14, 315 single testicular cells were profiled, and 10 cell-clusters were revealed, including spermatids, spermatocytes, SPG, Leydig cells, and Sertoli cells. In this study, due to the high cost of single cell sequencing, only one sample was sequenced, however, testes from five zebrafish were pooled for single cell sequencing to reduce the influence of individual variations. Our study not only illustrated new marker genes for each cell population of zebrafish testis, but also revealed the stronger paracrine influence of Leydig cells on Germ cells.

Furthermore, thousands of novel marker genes were identified, which could be important for further functional examination of each cell types in zebrafish testis. For example, sumo3b and stmn1a were discovered to be specifically expressed in SPG, *pimr93* and *si:ch73-367j5.3* were specifically expressed in the elongated spermatids. Recently, the adult testis transcriptome of ray-finned fish named orange-spotted grouper (*Epinephelus coioides*) was profiled at single cell level, and several novel marker genes were identified for testicular cells (Wu et al., 2021). Among the cell-marker genes identified, the genes such as *pprc1*, *top2b* were identified as the marker genes for spermatogonia and spermatocyte cells in both of orange-spotted grouper fish and zebrafish respectively. However, discrepancies had also been found in the two species, such as *supt16h* had been identified as top marker genes for spermatogonia cells in orange-spotted grouper fish but showed a broader expression pattern from spermatogonia cells to spermatids in our study (Supplementary Figure S3), which indicates cell type differential gene expression in the two types of fishes.

Next, based on the ligand-receptor paracrine interaction analyses, our study revealed stronger cell-cell communications between Leydig and germ cells, and the key players of LR interactions involved were also revealed in this study. For Sertoli cells, we only found 15 LR interactions between itself and SPG (SPG1 and SPG2), however, this number in Leydig is 206. It had been suggested that Sertoli cell is important for SPG stem cell niche by providing paracrine and other signals (França et al., 2016), which made us expect more LR interactions from Sertoli than from Leydig to SPG. To check possible factors that might increase Leydig-SPG LR interactions, we examined the influence of sequencing depth and the number of detected genes in Leydig and Sertoli for their paracrine interactions with SPG. Indeed, we found a higher average sequencing depth for Leydig (9,140 UMIs) compared to Sertoli (5,459 UMIs) (Supplementary Figure S1C), but the difference is not as large as the one for LR interactions (206 vs 15). Furthermore, Leydig and Sertoli cells contained similar number of average detected genes, which suggests its influence on the LR interactions might be small (Supplementary Figure S1D). This information indicates that both differences for sequencing-depth and the number of detected genes in the two somatic cell types cannot fully explain the observation of much stronger Leydig-SPG paracrine interactions in zebrafish testis, and future work is needed to study the paracrine influence of Leydig cells to the germ cells.

Finally, the transcriptome of the testes from other species had been extensively profiled at single cell levels, including human (Sohni et al., 2019; Guo et al., 2020; Guo et al., 2018; Shami et al., 2020), mouse (Chen et al., 2018; Green et al., 2018; Lukassen et al., 2018), monkey (Shami et al., 2020), and fly (Witt et al., 2019). However, as far as we know, before this study, there was no single cell level transcriptome study in zebrafish testis. Our study on the single cell RNA sequencing of zebrafish testes could be an important complementary resource for future study of spermatogenesis in zebrafish. Furthermore, to illustrate how the findings in zebrafish study could also contribute to human infertility studies, we examined the male infertility genes in GWAS Catalog database (https://www.ebi.ac.uk/gwas/)(Buniello et al., 2019). In total, we found nine genes that were recorded to be associated with “male infertility” or “non-obstructive azoospermia,” and three of them (*CLASP2*, *CRACR2A*, *SOX5*) were identified in this study as zebrafish testis cell-type specific marker genes (Supplementary Table S6). Notably, *clasp2*, *cracr2a* and *sox5* were identified as the marker genes for spermatocyte (c1), middle round spermatid (c6), and early round spermatid (c4) respectively in zebrafish testis (Supplementary Table S3), which indicates that zebrafish could be used as a model to study the genes that are associated with human infertility and might help to reveal the underlying mechanism.

## Data Availability

The datasets presented in this study can be found in online repositories. The names of the repository/repositories and accession number(s) can be found below: https://bigd.big.ac.cn/gsa, CRA003925.
