# Supplementary material for: Single Cell Transcriptome Sequencing of Zebrafish Testis Revealed Novel Spermatogenesis Marker Genes and Stronger Leydig-Germ Cell Paracrine Interactions
Source: Front Genet. 2022 Mar 11;13:851719. doi: 10.3389/fgene.2022.851719 (PMC8961980; doi:10.3389/fgene.2022.851719)

Supplementary Figures

Figure S1

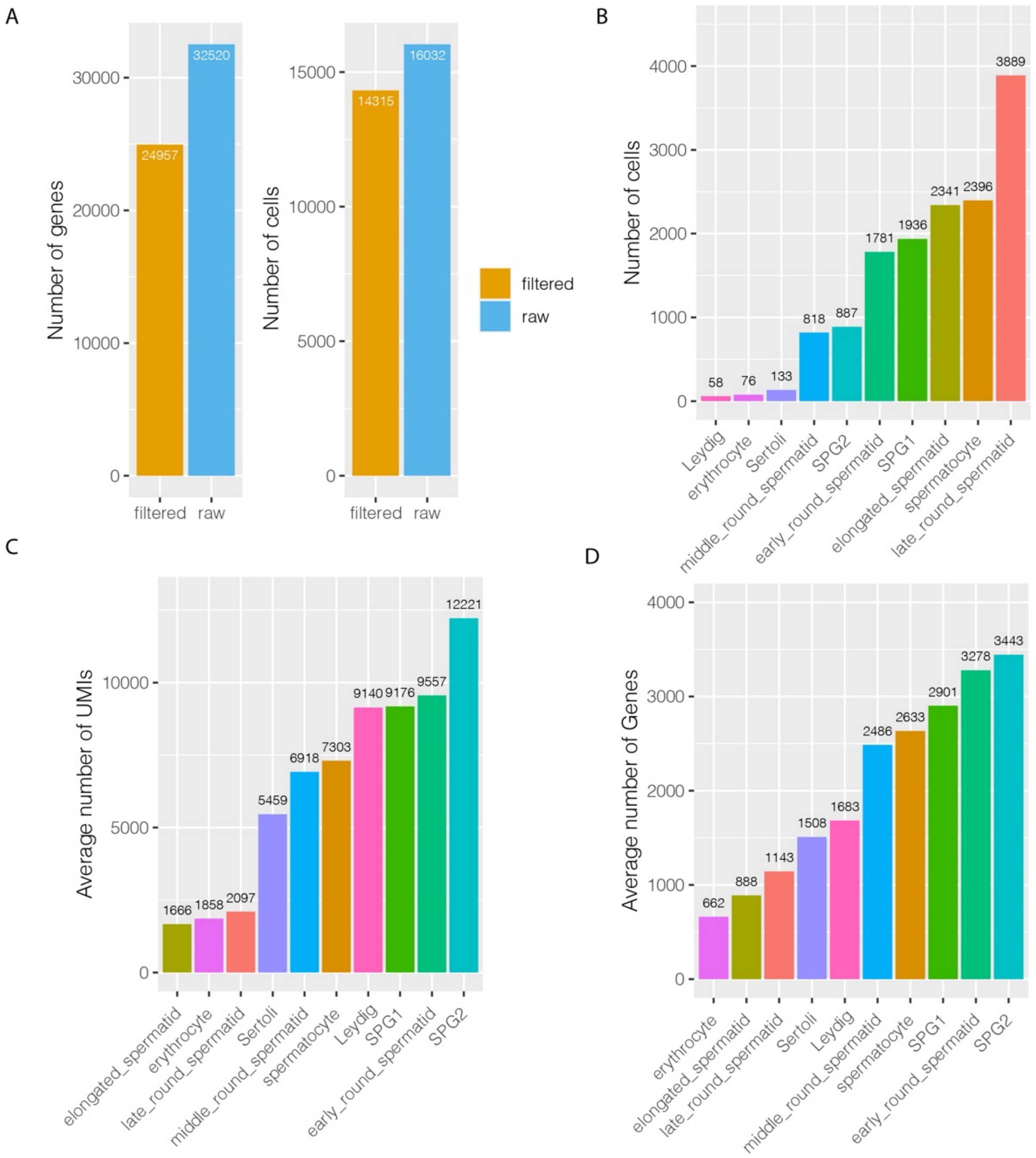

Figure S1. Basic summary about the single cell datasets. (A) Comparison of the number of cells and genes before and after filtering. (B) Number of cells detected in each cell type. (C)Average number of UMIs sequenced in each cell type. (D) Average number of genes detected in each cell type.

Figure S2: Expression of known marker genes.

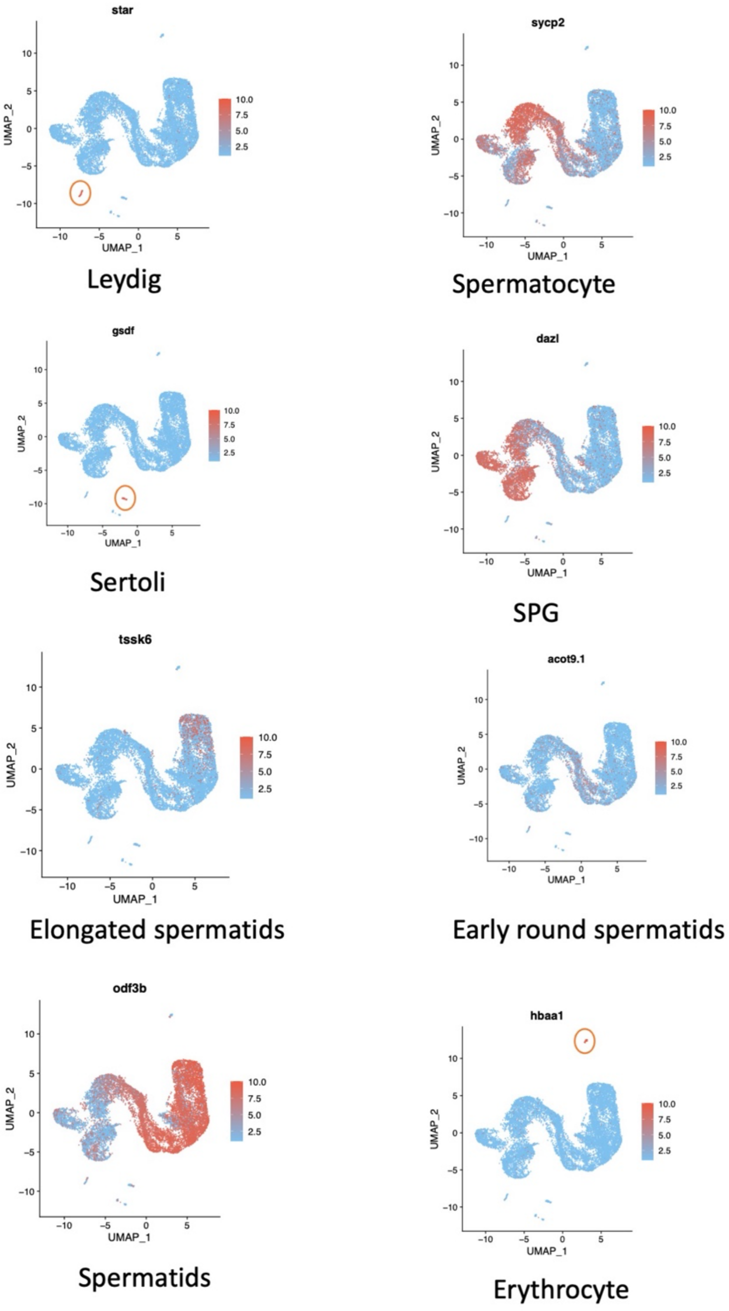

Figure S3: Expression of *supt16h*.

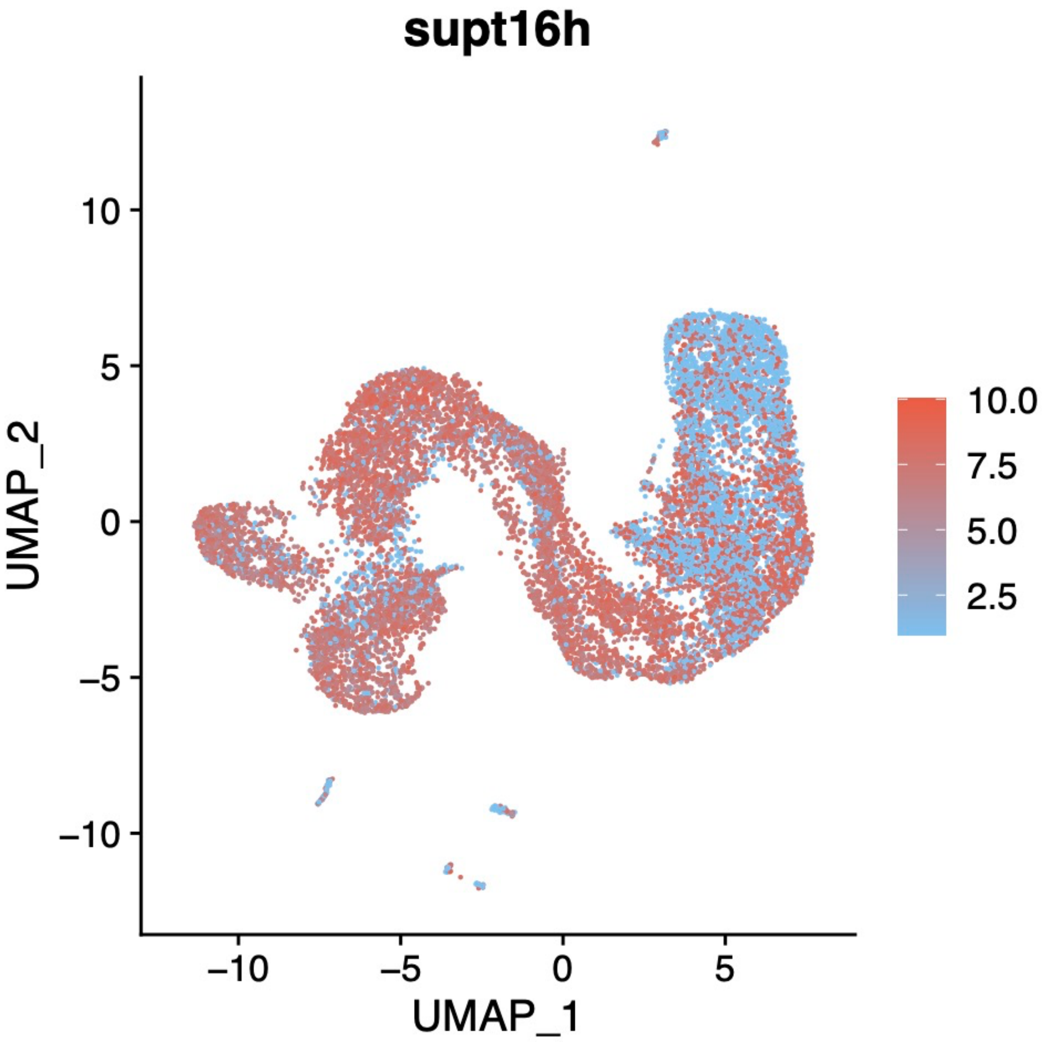

Supplement: Supplementary file 4 [file DataSheet1.pdf]
